# Differences in body composition, static balance, field test performance, and academic achievement in 10–12-year-old soccer players

**DOI:** 10.3389/fphys.2023.1150484

**Published:** 2023-03-30

**Authors:** Souhail Hermassi, Lawrence D. Hayes, Thomas Bartels, René Schwesig

**Affiliations:** ^1^ Physical Education Department, College of Education, Qatar University, Doha, Qatar; ^2^ Sport and Physical Activity Research Institute, School of Health and Life Sciences, University of the West of Scotland, Glasgow, United Kingdom; ^3^ Sports Clinic Halle, Center of Joint Surgery, Halle, Germany; ^4^ Department of Orthopaedic and Trauma Surgery, Martin-Luther-University Halle-Wittenberg, Halle, Germany

**Keywords:** physical performance, youth players, anthropometrics, body fat, body mass index

## Abstract

**Introduction:** This study aimed to compare 10–12-year-old Qatari male soccer players (*n* = 45) regarding different dimensions (anthropometric, academic and physical performance).

**Methods:** Anthropometric parameters (body mass, fat percentage (%BF), body mass index (BMI)) academic achievement (mathematics and science grade point average [GPA]) and physical performance [Yo-Yo Intermittent Recovery Test (level 1), squat jumps (SJ), counter-movement jumps (CMJ), stork balance test, 10 and 15 m sprint tests, T-half test for change-of-direction (CoD) ability, hand-grip strength, medicine ball throw (MBT)] were measured. Schoolchild soccer players were divided into three groups: 12-year-old players (U12; *n* = 16), 11-year-old players (U11; *n* = 14), 10-year-old players (U10; *n* = 15).

**Results:** Apart from mathematics, Yo-Yo IR1 and 10 m sprint, all performance parameters showed significant age effects. The largest age-related difference was observed for 15 m sprint (*p* < 0.001). Both adjacent age groups displayed significant differences for 15 sprint (U10 vs. U11: *p* = 0.015; U11 vs. U12: *p* = 0.023). Concerning academic performance, a significant age effect was found for science (*p* < 0.001). There was a main age effect on academic performance difference between U10 and U11 (*p* = 0.007). Academic parameters did not correlate with any physical performance parameter or anthropometric parameter. The strongest correlations were detected for body height and agility T-half test (*r* = −0.686) and medicine ball throw (*r* = 0.637). The biological maturity was strongly correlated with handgrip strength (r = −0.635).

**Discussion:** Soccer coaches and physical education teachers can use these data as reference values for evaluation of school-aged soccer players, and for ascertaining specific training targets. Obviously, short sprinting ability and aerobic capacity are not functions of age and need a specific training for significant improvements.

## Introduction

Soccer is simultaneously a physical and cognitive activity that has proven itself irresistibly popular with people all over the world (Stanković et al., 2023). Contemporary top-level soccer is marked by high-intensity activity throughout the entire match, also requiring a high level of a wider array of players’ functional and motor abilities (Tierney et al., 2016; Leyhr et al., 2021; Hermassi et al., 2022; Hermassi et al., 2022; Silva et al., 2022). Moreover, soccer is a multifaceted sport which comprises several activities (tackles, jumps, and direction, and speed changes) that tax numerous technical, cognitive, neuromuscular, and metabolic processes (Leyhr et al., 2021; Hermassi et al., 2022; Stanković et al., 2023).

Talent identification of soccer players is crucial for professional clubs as it is believed to increase the chances of a successful career as an elite player (McMorris et al., 2000; Perroni et al., 2018; Sarmento et al., 2018; O’Brien-Smith et al., 2020; Sørensen et al., 2022). The success of a team sport depends on various performance characteristics that are specific to the sport or transferable, such as physical attributes, technical and tactical skills, and psychological traits (Williams and Reilly, 2000; Leyhr et al., 2021; Hermassi et al., 2022). Profiling can be a useful tool for identifying talent, assessing strengths and weaknesses, determining player positions, and designing physical fitness programs (McMorris et al., 2000; Perroni et al., 2018; Sarmento et al., 2018; O’Brien-Smith et al., 2020; Sørensen et al., 2022). Studies have also found differences in physical, physiological, and anthropometric variables among different age groups, playing positions, and categories (Buchheit and Mendez-Villanueva, 2014; Figueira et al., 2018; Silva et al., 2022). Therefore, physical and anthropometric measurements, as well as motor tests, have been identified as important for predicting soccer performance (Buchheit and Mendez-Villanueva, 2014; Figueira et al., 2018; Bongiovanni et al., 2021).

Sport participation has benefits beyond the sport itself (Holt et al., 2012). For example, children who participate in activities tailored to their learning needs, such as learning-based sports, have better motor, situational, psychological, and social skills than those who participate in results-focused activities (Holt et al., 2012). Additionally, results-focused sporting activities may negatively impact learning processes (Manila, 2011). It is well established that tasks with clear goals, and ample opportunities for repetition and feedback lead to the best improvement. There is a growing body of evidence showing a positive relationship between academic achievement and physical fitness (Chomitz et al., 2009; Kwak et al., 2009; Wittberg et al., 2012), highlighting the importance of promoting physical fitness in adolescents to improve academic performance.

It has been reported children who exhibit superior physical fitness perform better academically (Chomitz et al., 2009; Kwak et al., 2009; Fedewa and Ahn, 2011; Wittberg et al., 2012). The most observed relationship with academic attainment is that of aerobic fitness. Less examined, or at least less likely to observe statistically significant relationships, are other measures of fitness like muscle strength or speed. This may be because the relationship between these parameters and academic attainment is less strong, and therefore less reported within the literature as a result of publication bias. It could also be that assessing muscle strength and speed is difficult to do in large cohorts, whereas the multistage fitness test can provide a proxy for aerobic fitness with many participants undertaking the test simultaneously. Whatever the reason, there have been calls for more research to examine the relationship between muscle function (i.e., strength and power) and academic attainment (Lago-Peñas et al., 2011; Kao et al., 2017). Another issue when considering the relationship between physical fitness and academic attainment is around conflating physical activity and fitness, as they are typically linked.

To date, previous reports confirm that correlations exist between fatness, physical fitness, and academic performance in pre-pubertal team sport in Qatar (Hermassi et al., 2021a; Hermassi et al., 2021b; Hermassi et al., 2021c). More research is required to test the different components of physical fitness (i.e., muscular strength, aerobic fitness, speed, agility, and postural stability) with academic performance (Haapala, 2013) in specific populations (i.e., soccer players). Using one population for this research question controls for physical activity levels to some extent, as all players within one team in a sport will likely complete the same amount of training.

In the literature, there is scant information concerning the physical fitness, body measurements, muscle function, and academic achievement of school-aged soccer players (Jonker et al., 2010; Hermassi et al., 2022). It is important to gather reference data on youth soccer players to evaluate their strengths and weaknesses and aid in talent identification. Standardized physical measurements can provide more accurate and useful information compared to subjective coaching evaluations (Hermassi et al., 2022). Therefore, the aim of this study was to evaluate the anthropometry and physical fitness of soccer players aged 10–12 years of age who attend school in Qatar. By comparing the anthropometric characteristics, sprinting, balance, change of direction, jumping, and aerobic performance of young soccer players of different age groups, it was hypothesized that there would be variations in these factors depending on age groups.

## Materials and methods

### Subjects

Following informed consent or assent prior to enrollment, 45 school children from a soccer academy in Doha, Qatar participated (age: 11.0 ± 0.8 years; body mass: 41.0 ± 7.3 kg; height: 1.46 ± 0.07 m; body fat: 14.8 ± 4.0%). Inclusion criteria were: 1) At least 3 years of playing experience; 2) not report any recent muscle or joint injuries; 3) soccer training 2–3 times a week for ∼4 h total time. Participants participated in weekly physical education lessons at school (∼45 min of mostly ball games) and avoided exercise for 24 h prior to testing.

This study was undertaken during in-season, from January to March 2021, and in accordance with the Declaration of Helsinki. In this context, the study was approved by Institutional Review Board of Qatar University (QU-IRB 1610-FBA/21).

### Experimental design

Testing was conducted on an outdoor soccer field at a specific time of day (17:00–19:00 h) to minimize the impact of diurnal variation. Participants were tested at least 3 days after a match. The study consisted of testing days to measure soccer-related physical performance characteristics (Hermassi et al., 2022). Dimensions and tests included were: sprinting performance (10 and 15 m), jumping performance (CMJ, SJ), throwing performance (medicine ball overhead throw), and aerobic capacity (Yo-Yo IR1).

Prior to each test, a general warm-up of 5 min low intensity running, 3 × 15 m progressive accelerations, and a maximal 20 m sprint was completed. Submaximal dynamic stretches and throws were also performed as previously described (Hermassi et al., 2021a). Tests were conducted over 5 days, in the same order for all participants (day 1: Anthropometry; day 2: Aerobic testing and stork balance test; day 3: Jumping tests; day 4: Sprinting tests and handgrip test; day 5: Agility T-half test). All tests (except for anthropometry, Yo-Yo IR1) were repeated after 2 weeks to enable test-retest reliability analysis (Table 1), with the second set of values being analyzed.

**TABLE 1:** Fitness test data from two sessions (*n* = 45). Descriptive statistics and intrarater reliability are presented for each test. Intraclass correlation coefficient (ICC) ≥ 0.75 and coefficient of variation (CV) ≤ 10% marked in bold. Data are reported as mean ± standard deviation (SD).

Test	Session one (mean ± SD)	Session two (mean ± SD)	ICC (95% CI)	CV (%) (95% CI)
10 m sprint (s)	2.27 ± 0.23	2.27 ± 0.21	**0.96** (0.93–0.98)	**2.8** (2.1–4.2)
15 m sprint (s)	3.38 ± 0.32	3.41 ± 0.32	**0.99** (0.98–1.00)	**0.9** (0.7–1.4)
Agility T-half test (s)	7.44 ± 0.91	7.78 ± 1.03	**0.93** (0.66–0.97)	**3.9** (3.0–6.0)
SJ (cm)	25.1 ± 6.37	25.6 ± 6.32	**0.98** (0.97–0.99)	**1.7** (1.3–2.6)
CMJ (cm)	28.0 ± 6.07	28.2 ± 5.99	**1.00** (0.99–1.00)	**1.6** (1.2–2.3)
Postural balance (s)	20.7 ± 8.63	20.5 ± 7.98	**0.99** (0.98–0.99)	**4.2** (3.2–6.5)
Medicine ball throw (m)	4.71 ± 0.67	4.74 ± 0.70	**0.99** (0.97–0.99)	**0.8** (0.6–1.2)
Grip strength (N)	15.9 ± 2.46	16.0 ± 2.54	**1.00** (0.99–1.00)	**1.7** (1.3–2.5)

### Variables

#### Anthropometry

All anthropometric measures were previously described (Hermassi et al., 2021a; Hermassi et al., 2022) and published open access. Standard techniques were used to determine mass, status, and BMI. The four-site skinfold method estimated % BF using Harpenden calipers and Womersley and Durnin, 1973 age-specific equations (1973).

#### Biological maturity

We estimated somatic maturity using proximity to peak height velocity (Y-PHV), and gender-specific equations which incorporate body mass, stature, leg length, and sitting height (Mirwald et al., 2002).

#### Physical performance

The Yo-Yo IR1 test was carried out following the procedure outlined by Krustrup et al. (2003). It involved 20-m shuttle runs at increasing speeds until participants were unable to continue, with 10-s breaks for active recovery in between. Trials were terminated if a participant did not reach the line in time twice in a row or gave up. From a standing start, participants sprinted 15 m in a maximal effort. Paired photocells recorded times at 10 m and 15 m. Photoelectronic cells were used to determine SJ and CMJ using contact time and flight time with a sampling rate of 1 kHz. To measure static balance, the Stork Balance Test (Miller, 2013) was used. Participants raised their heel off the floor on the “go” signal, balanced on one foot with the opposite foot against the inside of the supporting knee, with hands on their hips, and maintained this position for as long as they could. If the heel of the supporting leg touched the ground, or the foot moved away from the kneecap, the trial was terminated. A stopwatch was used to time each trial. T-Half test (Sassi et al., 2009) data were determined using electronic timing sensor. A standard adjustable digital hand-grip dynamometer (T.K.K. 5401, Tokyo, Japan) was used to determine handgrip strength of the dominant hand, with a sensitivity of 10 N. The best performance was taken for analysis. Medicine ball overhead throw (Negrete et al., 2010) were completed with a medicine ball with a weight of 3 kg and a diameter of 21.5 cm. All used physical performance tests are previously described in detail (Hermassi et al., 2022).

#### Academic performance

School records were used to assess academic performance, which was defined as the actual GPA and scores (ranging from 0 to 100) in mathematics and science as officially recognized in Qatar for the second semester of the academic year 2021–2022. Mathematics and science were included in the analyses because fitness is purportedly important for subjects more reliant on executive cognition, such as the subjects mentioned above, which in turn determines academic achievement (Hsieh et al., 2019; Hermassi et al., 2021a; Hermassi et al., 2022).

### Statistical analysis

Normal distribution (Shapiro-Wilk Test) and homogeneity of variances (Levene-Test) were conducted for all data to test for parametricity assumptions. Reliability was evaluated using intraclass correlation coefficients (Vincent, 1995) and coefficient of variation (CV) of pairs of intra-participant trials (Schabort et al., 1998). Descriptive statistics [median, mean, standard deviation (SD), minimum, maximum, and 95% confidence intervals (CI)] were calculated for all variables. Differences in anthropometric and performance parameters between age groups (U10, U11, and U12) were examined using Kruskal-Wallis H test and Mann-Whitney-U test for *post hoc* testing, as data were not normally distributed. Spearman’s rank-order correlations were used to evaluate the relationships between anthropometric (independent variables) and performance parameters (dependent variables). Magnitude of correlations (r) were interpreted as <0.1 = trivial, 0.1–0.3 = small, 0.3–0.5 = moderate, 0.5–0.7 = large, 0.7–0.9 = very large, and 0.9–1.0 = almost perfect (Cohen, 1988). A correlation of *r* ^2^ > 0.5 (explained variance >50%) was considered relevant and is marked in bold in the results sections. For a sample size of 45, the critical value for the product-moment correlation based on a two-sided *t*-test and alpha = 5% is *r* = 0.289 (Willimczik, 1997). A power calculation using nQuery Advisor 4.0 (Statistical Solutions, Saugus, MA) revealed that the study had 80% power to detect a mean difference of 3.0 cm in CMJ height (main outcome) with a sample size of 7 in each group using a two-sided *t*-test and *α* < 0.05, assuming a common standard deviation of 2.0 cm (Bortz, 1999). Statistical analyses were performed using SPSS version 28.0 for Windows (IBM, Armonk, NY, United States).

## Results

### Normal distribution and variance homogeneity

Twelve variables (body height: *p* = 0.049; body weight: *p* = 0.018; biceps skinfold: *p* < 0.001; triceps skinfold: *p* = 0.002; subscapular skinfold: *p* < 0.001; maturity offset: *p* = 0.031; sprint 10 m: *p* < 0.001; CMJ: *p* = 0.008; postural balance: *p* = 0.004; Yo-Yo IR1: *p* = 0.032; mathematics: *p* < 0.001; science: *p* < 0.001) were not normally distributed, and therefore median and arithmetic means are presented, for comparison of results with previous studies. Non-parametric tests evaluated differences between age groups.

### Intrarater reliability

All performance tests displayed excellent relative reliability (ICC ≥0.75). ICCs ranged from 0.93 (agility T-half test) to 1.00 (grip strength, CMJ). Furthermore, all variables displayed excellent absolute reliability with CVs <10% (Table 1).

### Anthropometric data

Anthropometric data by age group are displayed in Table 2, 3. Except for body height (*p* = 0.001) and body mass weight (*p* < 0.001), anthropometric parameters were not different between age groups at the *p* < 0.05 level (Table 2). The greatest differences were found for body mass (*p* < 0.001) with a significant difference between U11 and U12 (*p* = 0.037; Table 2).

**TABLE 2:** Demographic and anthropometric characteristics in relation to age groups.

	Body height (m)	Body mass (kg)	BMI (kg/m^2^)	Body fat (%)
	Median	Median	Median	Median
	Mean ± SD (95% CI)	Mean ± SD (95% CI)	Mean ± SD (95% CI)	Mean ± SD (95% CI)
**U 10** (*n* = 15)	1.41	33.0	17.3	14.3
	1.41 ± 0.05 (1.38–1.44)	36.5 ± 8.48 (33.3–39.8)	18.3 ± 3.53 (17.1–19.6)	13.4 ± 4.35 (11.4–15.4)
**U 11** (*n* = 14)	1.47	39.5	18.8	14.0
	1.45 ± 0.08 (1.42–1.49)	40.0 ± 5.70 (36.7–43.4)	18.9 ± 1.76 (17.6–20.2)	14.4 ± 3.18 (12.3–16.4)
**U 12** (*n* = 16)	1.51	45.0	19.9	15.7
	1.50 ± 0.04 (1.47–1.53)	45.9 ± 3.53 (42.8–49.1)	20.4 ± 1.18 (19.2–21.6)	16.4 ± 3.87 (14.4–18.3)
Kruskal-Wallis (*p*)	**p = 0.001**	**p < 0.001**	*p* = 0.053	*p* = 0.102
Significant differences between adjacent age groups (*p*)	-	**U11/U12: p = 0.037**	-	-

BMI, body mass index.

Significant differences marked in bold.

**TABLE 3:** Different skinfolds in relation to age groups.

	Biceps skinfold (mm)	Triceps skinfold (mm)	Suprailiac	Subscapular
			Skinfold (mm)	Skinfold (mm)
	Median	Median	Median	Median
	Mean ± SD (95% CI)	Mean ± SD (95% CI)	Mean ± SD (95% CI)	Mean ± SD (95% CI)
**U 10** (*n* = 15)	6.00	8.50	8.50	6.00
	6.50 ± 1.94 (4.92–8.08)	8.87 ± 3.26 (7.02–10.7)	7.87 ± 3.62 (6.10–9.63)	7.93 ± 5.01 (6.00–9.86)
**U 11** (*n* = 14)	7.50	9.25	7.00	7.25
	7.71 ± 3.16 (6.08–9.35)	9.93 ± 3.78 (8.02–11.8)	8.11 ± 3.23 (6.28–9.93)	7.07 ± 1.41 (5.07–9.07)
**U 12** (*n* = 16)	8.00	9.00	10.0	6.75
	9.06 ± 3.68 (7.53–10.6)	9.78 ± 3.59 (7.99–11.6)	10.3 ± 3.29 (8.57–12.0)	6.88 ± 3.65 (5.01–8.75)
Kruskal-Wallis (*p*)	*p* = 0.074	*p* = 0.679	*p* = 0.103	*p* = 0.707
Significant differences between adjacent age groups (*p*)	—	—	—	—

Significant differences marked in bold.

### Physical performance data

The best sprint performance was observed in the oldest age group (U12). For 10 m sprint (*p* = 0.456; Table 4) and Yo-Yo IR1 test (*p* = 0.116; Table 5) no significant age differences were observed. The largest age effect with significant differences between both adjacent age groups (U10 vs. U11 and U11 vs. U12) was detected for 15 m sprint (Table 4). We found significant performance differences between U10 and U11 (*p* = 0.015) and U11 and U12 (*p* = 0.023). Regarding adjacent age groups, the number of significant differences was larger for U11 vs. U12 (three significant differences: 15 m sprint, medicine ball throw, grip strength; Table 4, 6) than for U10 vs. U11 (only 15 m sprint; Table 4).

**TABLE 4:** Jumping and sprinting performance in relation to age groups.

	Jumping performance (cm)		Sprinting performance (s)	
	CMJ	SJ	10 m	15 m
	Median	Median	Median	Median
	Mean ± SD (95% CI)	Mean ± SD (95% CI)	Mean ± SD (95% CI)	Mean ± SD (95% CI)
**U 10** (*n* = 15)	32.0	27.0	2.31	3.38
	31.6 ± 1.64 (30.8–32.4)	27.3 ± 1.71 (26.2–28.3)	2.34 ± 0.13 (2.24–2.45)	3.39 ± 0.19 (3.31–3.47)
**U 11** (*n* = 14)	32.0	29.0	2.33	3.17
	32.6 ± 1.16 (31.8–33.4)	29.0 ± 2.22 (27.9–30.1)	2.31 ± 0.15 (2.20–2.42)	3.21 ± 0.13 (3.13–3.30)
**U 12** (*n* = 16)	33.0	30.5	2.20	3.10
	33.0 ± 1.51 (32.3–33.7)	30.0 ± 2.22 (29.0–31.0)	2.25 ± 0.28 (2.15–2.36)	3.05 ± 0.15 (2.97–3.13)
Kruskal-Wallis *p*)	***p* = 0.033**	***p* = 0.003**	*p* = 0.456	***p* < 0.001**
Significant differences between adjacent age groups *p*)	—	—	—	**U10/U11: *p* = 0.015**
				**U11/U12: *p* = 0.023**

CMJ, Countermovement jump; SJ, Squat jump.

Significant differences marked in bold.

**TABLE 5:** Aerobic capacity measuring in Yo-Yo IR1 and postural balance in relation to age groups.

	Aerobic capacity	Postural balance s)
	Yo-Yo IR 1 (m)	
	Median	Median
	Mean ± SD (95% CI)	Mean ± SD (95% CI)
**U 10** (*n* = 15)	760	21.0
	797 ± 76 (729–866)	19.0 ± 6.08 (15.4–22.6)
**U 11** (n = 14)	840	21.5
	837 ± 186 (766–908)	22.2 ± 8.22 (18.5–25.9)
**U 12** (n = 16)	920	30.5
	898 ± 116 (831–964)	27.5 ± 6.35 (24.0–31.0)
Kruskal-Wallis *p*)	*p* = 0.116	***p* = 0.005**
Significant differences between adjacent age groups (*p*)	—	—

Significant differences marked in bold.

**TABLE 6:** Agility, throwing performance and grip strength in relation to age groups.

	T-half test (s)	Medicine ball throw (m)	Grip strength (N)
	Median	Median	Median
	Mean ± SD (95% CI)	Mean ± SD (95% CI)	Mean ± SD (95% CI)
**U 10** (*n* = 15)	7.31	4.30	14.0
	7.42 ± 0.47 (7.02–7.81)	4.21 ± 0.51 (3.92–4.50)	13.9 ± 1.79 (13.0–14.8)
**U 11** (*n* = 14)	6.96	4.60	15.1
	6.85 ± 1.12 (6.44–7.26)	4.59 ± 0.51 (4.29–4.89)	15.3 ± 1.85 (14.4–16.2)
**U 12** (*n* = 16)	6.35	5.30	18.0
	6.48 ± 0.57 (6.10–6.86)	5.23 ± 0.62 (4.95–5.51)	18.2 ± 1.33 (17.4–19.0)
Kruskal-Wallis (*p*)	***p* = 0.005**	***p* < 0.001**	***p* < 0.001**
Significant differences between adjacent age groups (*p*)	—	**U11/U12: *p* = 0.010**	**U11/U12: *p* < 0.001**

Significant differences marked in bold.

Regarding academic performance (Table 7), a significant age effect was observed for science (*p* < 0.001). Academic performance was different between U10 and U11 (*p* = 0.007).

**TABLE 7:** Academic performance in relation to age groups.

	Mathematics	Science
	Median	Median
	Mean ± SD (95% CI)	Mean ± SD (95% CI)
**U 10** (*n* = 15)	90.0	90.0
	89.0 ± 8.28 (85.2–92.8)	87.8 ± 8.39 (84.8–90.8)
**U 11** (*n* = 14)	90.0	95.0
	90.4 ± 7.20 (86.4–94.3)	94.6 ± 4.14 (91.6–97.7)
**U 12** (*n* = 16)	95.0	97.5
	93.1 ± 6.55 (89.4–96.8)	97.2 ± 3.15 (94.3–100)
Kruskal-Wallis *p*)	*p* = 0.292	***p* < 0.001**
Significant differences between adjacent age groups (*p*)	—	**U10/U11: *p* = 0.007**

Significant differences marked in bold.

### Relationships between anthropometric, physical, and academic performance data

**FIGURE 1:**
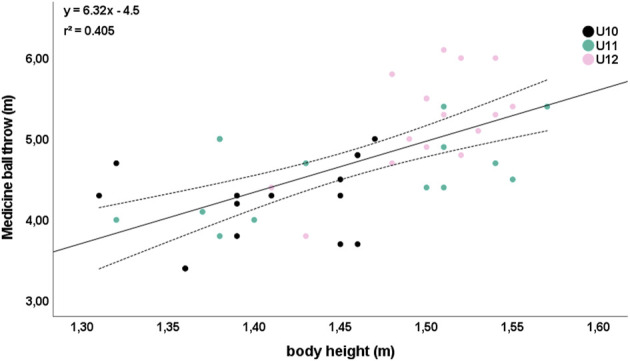
Relationship between body height and agility T-half test. Please note that one dot can represent several subjects.

**FIGURE 2:**
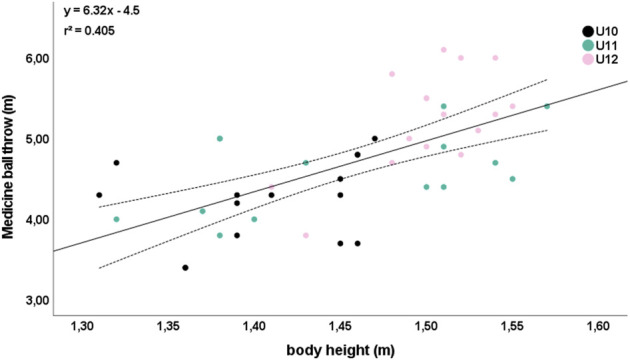
Relationship between body height and medicine ball throw. Please note that one dot can represent several subjects.

Meaningful correlations (*r* ≥ 0.5) were absent between anthropometric and academic performance. Five relevant relationships were observed between anthropometric and physical performance parameters. Particularly, body height was strongly correlated with agility T-half test (*r* = −0.692; Figure 1) and medicine ball throw (*r* = 0.668; Figure 2) independent of age.

Correlations were observed for body mass and agility T-half test (*r* = −0.519) and medicine ball throw (*r* = 0.596). The anthropometric parameter with the highest number (four) of relevant correlations with physical performance parameters was the maturity offset (level of biological maturity): 15 m sprint (*r* = 0.590); postural balance (*r* = −0.532); medicine ball throw (*r* = −0.560); and grip strength (*r* = −0.677).

## Discussion

The primary aim of the present investigation was to investigate the anthropometry, physical fitness, and academic achievement of 10- to 12-year-old Qatari male soccer players. Except for body height and body mass, anthropometric parameters were not significantly different between age groups. The largest differences between age groups were found for body mass between U11 and U12. Only for 10 m sprint and Yo-Yo IR1 were not significantly influence by age. The largest age effect with significant differences between both adjacent age groups was detected for 15 m sprint. Regarding adjacent age groups, the number of significant differences was larger for U11 vs. U12 (three significant differences: 15 m sprint, medicine ball throw, grip strength) than for U10 vs. U11 (for 15 m sprint only). Regarding academic performance, a significant age effect was found for science.

### Difference of anthropometric data

Several studies have reported anthropometric values like height and body mass are important factors in soccer performance (Cole et al., 2000; Reilly and Bangsbo, 2000; Arnason et al., 2004; Gravina et al., 2008; Perroni et al., 2014; Perroni et al., 2015; Perroni et al., 2016). Arnason et al. (2004) reported teams with higher fitness levels and lower body fat percentages had a higher league ranking. Our study found higher height, weight, and BMI values compared to those reported by Canhadas et al. (2010) for young Brazilian male athletes and higher BMIs than those reported by Gravina et al. (2008). Results observed herein were higher for height and weight compared to those reported by Di Luigi et al. (2006) in Italian soccer players, but lower for BMI in categories over 14 years. The differences may be due to maturational status favoring the U12 players, as noted by Malina et al. (2015). In this regard, it has been reported that a more advanced maturational status may lead to greater pubertal gains in height, body mass, and absolute and relative muscle mass (Malina et al., 2015).

Hermassi et al. (2020a) recently reported adolescent handball players from the Qatar handball first league exhibited 27% body fat, which is high for a young athletic population. The increase in sedentary lifestyles is leading to a rise in overweight adolescents in Qatar, along with a decrease in physical activity and motor skills (Hermassi et al., 2020a). A limited number of studies have assessed the anthropometric characteristics of youth soccer players especially concerning countries from the Gulf region (Dangour et al., 2002; Hermassi et al., 2022). The BMI of players participating in the present study was 19.2 ± 1.1 kg/m^2^, which is similar to values reported previously (Leyhr et al., 2021; Hermassi et al., 2022). Additionally, 4% (*n* = 2) of the present young soccer players had a BMI >25 kg/m^2^. The high BMI in the current study was unlikely solely due to a healthy muscle mass increase, and could have negative health effects.

### Difference of physical performance data

Sprint performance, along with other physical-fitness qualities crucial to soccer performance (e.g., aerobic fitness, explosive strength), is essential for success in the sport (Faude et al., 2012; Al Haddad et al., 2015). In young soccer players, strikers purportedly reach the greatest sprinting speeds during field testing, and these results confirm sprint performance is an important physical prerequisite for selection in young players (Al Haddad et al., 2015). As expected, for all parameters the peak sprint performance was observed in the oldest age group (U12). Only for the 10 m sprint were no significant age differences observed. The largest age effect between both adjacent age groups (U10 vs. U11 and U11 vs. U12) was detected for the sprint 15 m. The absence of differences in the 10 m sprint can be attributed to the small distance to differentiate velocity profiles (Marques et al., 2016). In this context, the 20 m and 30 m tests are more deterministic of soccer success (Marques et al., 2016). Marques et al. (2016) observed that the largest difference in sprint ability was between U14 and U16 players, rather than between U16 and U18 players. This finding may be related to peak physical maturation rates or alternatively as a result of limited upper body specific training in these age groups, given the pervasive concern over resistance training in youth athletes. This would be supported by the observation that adolescent boys who have advanced biological maturity tend to perform better physically (e.g., in speed, strength, power) than those who mature later (Lloyd et al., 2014).

In the present investigation, jump performance was comparable between age groups. Vertical jump performance is known to increase from U12 to U19 age categories (Gabbett and Herzig, 2004; Gabbett, 2009). Till and Jones (2015) demonstrated that players with greater maturity had superior vertical jump performance. These findings, coupled with the large inter-individual variability emphasizes the importance of tracking improvement of jump performance and strength characteristics of individuals. Reasonably, lower limb power could be the reason for improvements of CMJ with age, as a result of neural and muscular adaptations with age (Silva et al., 2022).

Success in soccer demands not only change-of-direction skills but also strong perceptual and decision-making abilities, demonstrated through advanced anticipatory motor performance (Pojskic et al., 2018). Hence, agility has been proposed as a key performance indicator and a necessary component of standard physiological testing for soccer players (Pojskic, et al., 2018). Fiorilli et al. (2017) reported U12 soccer players performed worse in agility tests [e.g., direction speed (CODS) and reactive agility (RA)], reasoning they were unable to carry out the perceptual and decision-making components of agility rapidly and respond with adequate movements, compared to their older counterparts.

Elite soccer players need to execute frequent high-intensity actions, making well-developed intermittent aerobic fitness crucial (Rowland, 1997). The current study found similar Yo-Yo IR1 performance between groups, though substantial variation within each group may have masked differences that did reach the *p* < 0.05 level (Scott et al., 2017). Considering anaerobic energy systems are improved after peak maturation (Selmi et al., 2020), these systems could aid anaerobic speed reserve as well (Selmi et al., 2020).

### Academic performance

Regarding academic performance, a significant age effect was found for science. The main age effect based on the performance difference between U10 and U11. Numerous psychosocial constituents ostensibly explain the role of physical fitness in academic attainment (Hermassi et al., 2021b). A large body of research has considered the extent to which educational performance predicts later-life body mass, and much research also focuses on the reverse causative relationship, concerning how body mass affects educational outcomes (Hermassi et al., 2021a; Hermassi et al., 2021b; Hermassi et al., 2022). Physical fitness is typically correlated with improved global health, which associates with academic achievement in young athletes (Hermassi et al., 2020b; Hermassi et al., 2021a; Hermassi et al., 2022).

Scientists studying sports have integrated this knowledge of brain functioning, using it to explain the contribution of physical exercise and how cognitive performance may increase performance in certain facets of sport (Fink et al., 2018; Verburgh et al., 2014). However, sport practice is associated with cognitive abilities (e.g., memory, executive function, attention, processing speed, and language processing) in adolescents (Huijgen et al., 2015; Anderson et al., 2019; Hermassi et al., 2021c). Appropriate cognitive functioning permits adaptation environments and appropriate psychosocial development and mental health (Huijgen et al., 2015; Anderson et al., 2019; Johnson et al., 2017). Cognitive performance is seemingly plastic, and recent meta-analytical evidence from 20 studies reports improved cognitive performance across several domains following body mass loss interventions (Veronese et al., 2017). Several studies have intimated that children who are more physically active exhibit better academic attainment than those who are physically inactive (Fedewa and Ahn, 2011; Hermassi et al., 2021c).

Studies have examined cognitive functions or motor skills separately in relation to success in top-level soccer or high performance in general (Lovecchio et al., 2021). At the youth level, physical skills are a strong indicator of a talented soccer player among various neuro-motor abilities (Sassi et al., 2009; Rommers et al., 2019). To profile players effectively, it is crucial to assess these multi-faceted skills for talent identification, not just selection. Talent selection is based on current abilities, whereas talent identification is a long-term approach that ensures maximum athletic and psychological potential is reached.

The present study supports previous cross-sectional findings which have demonstrated fitter students exhibit superior academic achievement (Hermassi et al., 2021a; Hermassi et al., 2021b). Explanations for this phenomenon are likely multi-factorial, and one may be that physical fitness is associated with superior overall health, in turn correlating with academic attainment (Lovecchio et al., 2021). Greater physical fitness leads to improved classroom behavior and attention (Esteban-Cornejo et al., 2014). Lovecchio et al. (2021) emphasize both components (cognitive and motor skills) result in successful performance in soccer among players in middle childhood (Vestberg et al., 2017). Thus, it could be expected that superior academic attainment could be associated with superior team sport performance.

### Limitations

A measure of (or proxy of) sexual maturity was absent in this study which is a limitation as different growth spurt timings could influence body composition and relationships between body composition and fitness. Moreover, body composition compartments have their own temporal deviations. Regardless, participants in this study were of a narrow age range, which attenuates this limitation to some degree. Non-etheless, future investigations could incorporate sexual maturation assessment when exploring relationships between anthropometry and physical performance in young team sport athletes. A secondary limitation is that since the study was cross-sectional, and therefore attributing causality is impossible.

## Conclusion

This investigation provides valuable data to evaluate the physical and academic performance of young male soccer players in a valid way. Especially, the dependence on age, the interaction between different dimensions (anthropometric, physical, and academic performance) and the longitudinal development over 3 years are very important aspects. These findings can inform coaches and sport scientists involved in training or testing young male soccer players. Further studies are required to explore optimal training approaches and content to effectively develop speed-related performance in young soccer players. Yet, early specialization may be harmful for talent development, and a “well-rounded” individual may outperform those with specialized training later in maturity. Therefore, research in the youngest players may provide the basis for a promising, long-term soccer development model and talent identification.

## Data Availability

The original contributions presented in the study are included in the article/supplementary material, further inquiries can be directed to the corresponding author.
